# Mesenteric ischaemic occurring in conjuction with acalculous cholecystitis: a case report

**DOI:** 10.1186/1757-1626-1-366

**Published:** 2008-12-01

**Authors:** Teegan Lim, Benjamin HL Tan, T Reuben Pepple, Nerukav Radhakrishnan, Samir Afify, Regi George

**Affiliations:** 1 Department of Gastroenterology, Rochdale Infirmary, Whitehall Street, Rochdale, OL12 0NB, UK; 2 Clinical and Surgical Sciences (Surgery), University of Edinburgh, 51 Little France Crescent, Edinburgh, EH16 4SA, UK; 3 Department of Surgery, Rochdale Infirmary, Whitehall Street, Rochdale, OL12 0NB, UK

## Abstract

**Background:**

The incidence of mesenteric ischaemia is rising possibly due to increasing awareness and early diagnostic tools available. However it remains a challenging diagnosis especially in the elderly population.

**Case report:**

We report an unusual case of acute mesenteric ischaemia in an elderly lady occurring in conjunction with acalculous cholecystitis. A 71 year old woman was referred to our hospital with abdominal pain, vomiting, diarrhoea and pyrexia. An initial ultrasound scan of the abdomen revealed acute acalculous cholecystitis.

**Conclusion:**

She failed to respond to medical treatment and further investigations revealed concurrent mesenteric ischaemia.

## Background

Mesenteric ischaemia results from decreased blood flow to the bowel resulting in cellular injury as a result of decreased oxygen and nutrient supply. It can be classified temporally (acute vs. chronic), by location (small bowel vs. colonic) and by vessel (artery, arteriole, vein, venule) involved [1]. Although acute mesenteric ischaemia (AMI) accounts for only about 1–2% of gastrointestinal illnesses [2], diagnosis remains a challenge and is often delayed especially in the elderly who commonly present with vague and non specific symptoms [3]. We describe an unusual presentation of AMI, in a 71 year old woman, occurring in conjunction with acalculous cholecystitis.

## Case presentation

A 71 year-old Caucasian lady was referred to our hospital with a one week history of diarrhoea, vomiting, epigastric pain and pyrexia. She was obese with a BMI of 38.3 but no significant past medical history. She had been to Australia a month prior but was clinically well during her stay there. Blood inflammatory markers revealed a normal white cell count but a raised C-reactive protein (CRP) of 54 mg/l. Liver function tests were unremarkable apart from a raised serum alanine transaminase (ALT) of 70 U/l. Stool and blood cultures were negative. Clostridium difficile toxin was not detected. She was treated supportively for infective gastroenteritis and her symptoms resolved spontaneously after 3 days. She was subsequently discharged and an outpatient abdominal ultrasound was arranged to investigate the cause of the mildly raised ALT.

A week later, she was developed severe upper abdominal pain, rigors and pyrexia of 39°C and was readmitted to hospital. The abdominal ultrasound showed a thickened and inflamed gall bladder with no calculi seen. Inflammatory markers on this admission showed an elevated white cell count of 11 × 10^9^/l, and an increased CRP of 72 mg/l. Liver function tests continued to be unremarkable apart from ALT which remained elevated at 80 U/l. Abdominal and chest X-rays were unremarkable. Arterial blood gases were normal. She was diagnosed with acalculous cholecystitis and treated with intravenous broad spectrum cephalosporins. Unfortunately her condition did not improve and she subsequently developed diarrhoea and bleeding per rectum.

Further investigations were then carried out including a flexible sigmoidoscopy which was unremarkable apart from diverticular disease in the sigmoid colon. A computed tomography (CT) scan of the abdomen showed moderate small bowel dilatation and some enhancement of the bowel wall, which raised the possibility of bowel ischaemia. Thrombosis of the proximal superior mesenteric vein was also noted.

An exploratory laparotomy was then performed and a 17 cm segment of the jejunum was found to be ischaemic and gangrenous. The affected section of small bowel was resected followed by an end to end anastomosis. Histology of the resected specimen confirmed full thickness infarction of the small bowel associated with thrombi in the mesenteric vessels, the overall appearance of which was consistent with acute mesenteric ischaemia. There were several thrombi within the mesenteric vessels, raising the possibility of embolic aetiology.

## Discussion

AMI is a potentially fatal abdominal emergency with a mortality rate of 60 – 80% [4,5]. Although uncommon, its incidence is increasing [6]. Risk factors include atherosclerotic disease, congestive heart failure and other low-output states, arrhythmias, valvular disease, recent myocardial infarction and hypotension. In patients older than 45, approximately one half have some degree of atherosclerosis of the celiac, superior, and inferior mesenteric arteries [7]. The superior mesenteric artery most commonly is implicated in AMI.

Acalculous cholecystitis, i.e. inflammation of the gallbladder without evidence of calculi or sludge, comprises 2 – 15% of all cases of acute cholecystitis [8]. The pathogenesis of acalculous cholecystitis is poorly understood but one of the common postulated theories involves ischaemia [9]. There have been two cases reported of patients who developed acute acalculous cholecystitis without apparent risk for the disease other than severe visceral atherosclerosis [10]. In addition, Warren noted that multiple arterial occlusions, with absent or minimal venous filling, were consistent features of acute acalculous cholecystitis [11].

The patient's initial symptoms were vague and did not provide evidence to suggest mesenteric ischaemia which is not uncommon in elderly patients [3]. Initial radiographic investigation of choice was an abdominal ultrasound, of which the only pathology revealed was acalculous cholecystitis. Only ineffectiveness of initial treatment and progression of symptoms prompted further and more detailed investigations which revealed infarcted small bowel requiring surgical intervention.

To the best of our knowledge, this is the first case of acute acalculous cholecystitis occurring with AMI. It is highly plausible that widespread ischaemia may have contributed to both conditions.

## Consent

Written informed consent was obtained from the patient for publication of this case report and accompanying images. A copy of the written consent is available for review by the Editor-in-Chief of this journal.

## Competing interests

The authors declare that they have no competing interests.

## Authors' contributions

TL, BT and TP collected the data and drafted the initial manuscript. SA operated on the patient with the assistance of TP. NR and RG contributed to the final approval and revision of the manuscript. All authors read and approved the final manuscript.
